# Considerations for an urban health perspective in Chile from the “Quiero Mi Barrio” program

**DOI:** 10.11606/s1518-8787.2023057004264

**Published:** 2023-03-15

**Authors:** Paola Olave-Müller, Natalia López-Contreras, Patricio Alvarez Kocher, Paola Jirón, Camilo Bass del Campo, Soledad Burgos de la Vega

**Affiliations:** I Universidad de La Frontera Facultad de Medicina Departamento de Salud Pública Temuco Chile Universidad de La Frontera. Facultad de Medicina. Departamento de Salud Pública. Temuco, Chile; II Universidad de La Frontera Unidad de Vicerrectoría Investigación y Postgrado Temuco Chile Universidad de La Frontera. Unidad de Vicerrectoría Investigación y Postgrado. Temuco, Chile; III Universitat Pompeu Fabra PhD Programme in Biomedicine Barcelona Spain Universitat Pompeu Fabra. PhD Programme in Biomedicine. Barcelona, Spain; IV Universidad Austral de Chile Facultad de filosofía y humanidades Escuela de postgrados Valdivia Chile Universidad Austral de Chile. Facultad de filosofía y humanidades. Escuela de postgrados. Valdivia, Chile; V Universidad de Chile Facultad de Arquitectura y Urbanismo Santiago Chile Universidad de Chile. Facultad de Arquitectura y Urbanismo. Santiago, Chile; VI Universidad de Chile Escuela de Salud Pública Santiago Chile Universidad de Chile. Escuela de Salud Pública. Santiago, Chile

**Keywords:** Social Determinants of Health, Urban Renewal, Community Participation, Neighborhood Characteristics, Qualitative Research, Chile

## Abstract

**OBJECTIVES:**

To explore the perceptions of residents regarding their health and well-being in areas of personal and collective life, in relation to the experience of urban transformation originated by the Program for the Recovery of Neighborhoods in Chile “Quiero mi Barrio” (PQMB).

**METHODS:**

Qualitative study conducted in eight neighborhoods, which were subject to interventions between 2012–2015, located in seven communes of Chile: Arica, Renca, Padre Las Casas, Villarrica, Castro, Ancud. Eighteen focus groups and 27 interviews were conducted between 2018 and 2019. A content analysis was carried out following the social determinants of health approach.

**RESULTS:**

Material conditions of neighborhood infrastructure and psychosocial determinants were the main emerging and predominant categories in the residents’ narratives. The new or improved infrastructure enhances sports and playing practices, as well as contributes to the feeling of safety and to the improvement of walkable spaces, support networks, socialization and dynamization of social organization. However, neglected aspects were visualized. The program had limitations of structural character that operate locally, such as aging, individual lifestyles that limit participation, and contexts of insecurity, especially in neighborhoods victims of drug trafficking.

**CONCLUSIONS:**

The urban changes originated by the PQMB included improvements in neighborhood infrastructure and in the psychosocial environment, which are perceived by residents as beneficial aspects and promoters of collective wellbeing. However, global phenomena, and those related to the program, limit its scope and have repercussions on the perception of overall wellbeing of the residents in the neighborhoods. To go deeper into how this or other state neighborhood programs may or may not favor equitable access of different social groups, or which works may be better used by the groups, is an aspect that enhances the integral action with other sectors and local actors in the territories.

## INTRODUCTION

In several Latin American and European cities, residential segregation and socioeconomic inequality have affected residents’ quality of life^1,2^. Among Latin American countries, Chile has reached urbanization levels as high as 87.8%, according to data from its 2017 Census, and felt the same global phenomena of residential segregation and socioeconomic inequality which brought territorial implications^3^ and deteriorated urban-rural living conditions.

In 2000, 2006, and 2016, the National Quality of Life Survey, conducted by the Chilean Ministry of Health^4^, found that its communities lacked infrastructure and public security and showed a weak connection between citizens and social organizations, key entities to construct healthy spaces for good living. In 2018 and 2020, governmental bodies and organizations linked to urban planning created microscale indicators to evaluate national areas, many of which showed instable environmental accessibility and infrastructure, crucial dimensions to guarantee their populations a sense of equity, justice, and well-being^5^.

Along with the increase in chronic non-communicable diseases^6^, healthcare in Chile has promoted the social determinants of health (SDH) approach since 2005, seeking well-being and health equity^7^ and producing guidelines to find the structural dimensions of health inequality, including the conditions “in which life takes place”^8^. Health initiatives, undertaken in 2009^9^, addressed several urbanization problems, privileging a territorial approach aimed at integrating SDH into health agendas and avoiding achieving continuity or projecting health policies articulated with other sectors.

On the other hand, residential segregation and its social effects have also occupied a central space in the design of strategies^10^within the recent framework for public policies on housing and urban planning in Chile. The Chilean Ministry of Housing and Urban Planning created the neighborhood recovery program “Quiero Mi Barrio” (PQMB) (*I Love My Neighborhood Program* in Spanish) in 2006. By 2017, it had promoted interventions in 520 neighborhoods across 15 regions, conducting 3,691 works and benefitting 1,132,714 residents. It currently aims to “improve quality of life in neighborhoods dealing with social segregation and/or vulnerability or varying degrees of urban deterioration, including them in a participatory, comprehensive, and viable process of local urban regeneration”^11^. Although the core principles of the Program do not make explicit their interest in influencing “health” of population, its design directly affects what the health sector consider intermediate SDH, including participation in social groups and improving local urban resources and services.

To favor integrative processes of public policy initiatives, this study aimed to explore residents’ perceptions of their health and well-being in personal and collective life areas in relation to urban transformation experiences stemming from the Chilean program to recover neighborhoods “Quiero mi Barrio” (PQMB). This study provides an organizing and reflexive framework to highlight the benefits and limitations resulting from the neighborhood recovery process to broaden its understanding and recognize the scope of these actions to produce urban spaces.

## METHODS

This qualitative study was conducted between 2018 and 2019 and adopts the SDH approach to assess areas of results^12^, deeming them as large domains of constraints on the circumstances or situations in which “people are born, grow up, live, work, and grow old”^13^ and in which dominant political, economic, and social actions have strong repercussions to produce and reproduce social health inequality. Our sample consisted of eight neighborhoods which received interventions beginning between 2011 and 2012 and ending three to four years later. To select neighborhoods for analysis, four administrative regions were classified according to their geography, climate and population density to incorporate heterogeneous elements into our sample, such as being in an area with high migratory exchange, strong dissatisfaction with physical and social environments, social isolation, conflictivity and fragility political-territorial. In this study, neighborhoods were chosen based on the beginning of interventions (all same year) and feasibility of access (contacts, referents). The available documentation was analyzed to assess each neighborhood and synthesize the conducted interventions (Chart 1).

**Chart 1:** Characteristics of the chosen neighborhoods and performed interventions.

Implementation of the PQMB			
Neighborhoods (city and region) and year of implementation of the PQMB	History of the neighborhood	Project management	Social management
**Norte Grande/Ignacio Serrano **(Arica, Region of Arica and Parinacota)	Its origin is based on the gradual urbanization of the division corresponding to “Población Chile” in the mid-1960s. Neighborhoods joined territorially	Construction of a park.	Activities of associativity- training for leaders oriented (A); Appropriation and use (B); Neighborhood coexistence (C).
(2012–2014)	**Population Norte Grande:** 1.802 inhabitants.		
	**Population Ignacio Serrano:** 1.758 inhabitants.		
**Lo Velásquez Norte/Sur** (Renca, Metropolitan Region)	Its origin stems from the construction of social housing in the 1980s (1984–1986) as part of the urban expansion of Santiago. Context of social and criminal unrest.	Construction of a multipurpose field, community center, improvement of public lighting and squares. Urban improvement.	Activities of Associativity training for leaders oriented (A); Appropriation and use - neighborhood identity and environmental plan oriented (B); Neighborhood coexistence (C).
(2012–2015)	**Population Lo Vásquez Norte:** 2.327 inhabitants		
	**Population Lo Vásquez Sur:** 2.163 inhabitants.		
**Diego Portales ** (Villarica, Region of La Araucanía)	It began as a private land occupation in the 1970s, regulated in the 1980s. Showed a context of social uprooting between sectors in the neighborhood.	Construction of bus stops, recovery of a multipurpose field, green areas, and squares. Construction of a community center.	Activities of Associativity- training for leaders oriented (A); Appropriation and use (B); Neighborhood coexistence (C).
(2012–2015)	**Population:** 2.700 inhabitants.		
**Pulmahue** (Padre Las Casas, Region of La Araucanía)	Its origin is based on the construction of basic housing awarded by SERVIU subsidies at the start of the 2000s. Showed a perception that insecurity decreased as activities brought neighbors together.	Recreational and physical conditioning stations.	Activities of Associativity training for leaders oriented (A); Appropriation and use (B); Neighborhood coexistence (C).
(2012–2015)	**Population:** 2.144 inhabitants.		
**Bellavista** (Ancud, Región of Los Lagos)	Its origin stems from the construction of social housing in the 1990s due to an increase in the fishing industry. Showed a context of inadequate public services and lack of employment and opportunities.	Construction of viewing platforms, parks, and an open-air multipurpose field; improvement to another multipurpose field. Construction of a community center and pedestrian walkway.	Activities of Associativity- training for leaders and neighborhood visits (A); Appropriation and use - oriented neighborhood identity (B); Neighborhood coexistence (C)
(2012–2015)	**Population:** 1.675 inhabitants.		
**La Isla ** (Castro, Region of Los Lagos)	Its origin is based on the construction of social housing between the end of the 1980s and the beginning of the 1990s. Context of neighborhood spaces and green areas with neither appropriation nor care.	Construction of two squares, a viewing platform, community civic center; improvement of covered multipurpose playing field. Construction of pedestrian walkway.	Activities of Associativity- training for leaders (A); Appropriation and use oriented to socio-educational activities (B), Neighborhood coexistence (C)
(2012–2015)	**Population:** 1.543 inhabitants.		

(A) training and reinforcement of functional organizations in the neighborhood; interregional community encounters between neighborhood leaders

(B) activities tending to strengthen identity and belonging to the neighborhood; organization of practices and cultural manifestations in community spaces in the neighborhood; promotion of waste management; promotion of the efficient use of basic resources

(C) implementation of mechanisms to mediate the community in conflict resolution at the leadership and neighborhood level; reconversion of residual public, inaccessible, and unsafe spaces for community use.

Source: Prepared by the author based on reports from the Chilean Ministry of Housing and community reports

To obtain data, 18 focus groups (FG) were structured and 27 semi-structured interviews (I), by a team of anthropologists. A cumulative theoretical sampling was used with a saturation criterion^14^. The following criteria were established to structure FG and conduct I: older adults with and without a social role in their community (aged ≥60 years), young people (aged 15-20 years), and women with preschool-aged children and emerging profiles. During the interviews, aspects of structuring processes, changes perceived in neighborhoods, and assessments of these changes in health and collective well-being were addressed. FG and I were recorded, transcribed, and manually analyzed via open categories to reduce, organize, and structure data by analogy around opinions and substantial ideas which were common to groups or showed divergences with a greater descriptive than interpretative focus, according to SDH macrocategories^15^. Results were triangulated by the different researchers collaborating in this study.

This study was approved by the Ethics Committee of the Faculty of the Universidad de La Frontera (Temuco, Chile) for studies on human beings. All interviewees agreed to participate.

## RESULTS

We organized our results into two highly interrelated neighborhood constraints predominating in residents’ accounts: a) material and b) psychosocial categories. We describe both the perceived beneficial aspects of interventions and their overlooked or limiting aspects for communities’ well-being.

### a) Material constraints in neighborhoods (Charts 2 and 3)

**Chart 2:** Contributions by the Quiero mi Barrio Program (PQMB) to residential well-being.

Residential surroundings	Use of the infrastructure created for sports and recreation	“I see children who are 7 to 5 years old more or less on the playground equipment. The older ones use the playing field, they go there to play ball. They come from Camilo to use the playing field, the one they have behind their community center isn’t used much” (Teenage girl, Barrio La Isla)
		“(the park has been good because we find) people enjoying the sun, talking, older adults sit there, they are chatting about their things and all that; A good use has been made of this avenue” (Migrant, Barrio Norte Grande)
	Recycling of illegal dump sites and improvement of community practices	“We installed a multipurpose field and we put lighting behind the community center, and now the neighbors use it as a parking lot so there are no more dump sites” (President, Neighborhood Association, Barrio Bellavista).
		“Before, they left garbage on the corner but three months ago the neighbors got together and talked about it and now very Saturday they collect the trash and have to throw it there (container), it improved the issue we had with garbage” (Young men, Barrio Ignacio Serrano).
	Fitting out of paved spaces	“I find that I feel good in my neighborhood now that everything is paved, it is pleasant. From time to time, there are occasions where I stand at the door with my husband, if we don’t sit (outside) to watch the hustle and bustle because it’s nice, because there are no gusts of wind with dust, that wind that used to come with sand…” (Older Lady, Barrio Ignacio Serrano)
Housing conditions and related resources	Support for external projects	“With this (complementary external project), we were able to improve some of the houses owned by old people in the population (…). Then, arranging for us to be part of a neighborhood program, it supported us in neighborhood association projects” (President, Neighborhood Association, Barrio Bellavista).
	Perception of added values to houses	“And we changed the face of it (the neighborhood), we also added value to the houses because, with a park nearby, it also raises that” (Neighborhood leader, Ignacio Serrano)

**Chart 3:** Aspects of the residential surroundings negatively associated or pending with the “Quiero mi Barrio” program.

Residential surroundings	To address the historical deterioration of streets and sidewalks	“For example, my dad has a problem with his leg and the sidewalks are in poor condition … he can suddenly fall and things like that. Likewise, the streets are narrow, and the cars pass by, some pass by really fast” (Teenage girl, La Isla)
		“Over time, the sidewalks were damaged, and we complained several times that the sidewalks had to be incorporated, no… no, they didn’t add them to that project (PQMB)” (President, Older Adults)
	Deterioration or destruction of works created by the PQMB	“It is not possible; it is all damaged…here without going farther. I passed by here on the way to the hospital, how many hundreds of bottles have been smashed here? Then, who’s going to bring a child here to play? If he falls, he will get really hurt, but nothing would happen to the stones and the field…” (Young man, Barrio La isla)
		“If you pass by there now you see that some youngsters were messing around for fun the other day and they put huge holes in the backboards… Sure, the backboards where the hoops go, and they stole all of that too, and the field back there is also messed up and dirty (Older Lady, Barrio Lo Velásquez)
	Undesired use of remodeled spaces	“We have playground equipment, people sometimes go there to exercise during the day but at night an outsider group comes to drink and play music, and sometimes (typically on Saturdays) they start at like one in the morning to play music and a group comes and they start dancing and messing around” (Mother, Barrio Lo Velásquez Norte)
	Displacement of commerce and interruption of labor practices (Norte Grande)	“But this (newly built park) also covered up everything, it blocked the view, it prevented us from being seen and make more sales. Before I took my bath, my mom would make breakfast and we would sell, like, a lot. (…) because when the open-air market was making money, we did what we needed to sell, we sold breakfast (…) and it made me good money (…)” (Older man, Barrio Norte Grande)
Housing conditions	Individual maintenance costs (PQMB ignores home improvements)	“I have made repairs, made more rooms there because it was just this, there was a bedroom, kitchen and bathroom, and nothing else … Yes, (I have been gradually fixing it) in the improvements I have applied for” (Older man, Barrio Pulmahue)
		“I did all the other stuff too, put ceramic on the floor, everything, we bought it little by little, got it together and then called the tradesman” (Older man, Barrio Ignacio Serrano)

Participants perceive PQMB as playing a relevant role in improving the infrastructure of their neighborhoods, promoting new practices, uses of public space, and a positive perception of the area. They find that the construction of playgrounds and parks constitutes spaces which promote physical activity and play among young people and children and social meetings among neighbors. Removing illegal dump sites emerges as a direct PQMB action as well as an indirect one due to other interventions, such as lighting and reconverting spaces for community use. Interviewees also indicate that the spaces PQMB reconverted into public circulation areas, with lit and decorated sidewalks, provide residents with greater security. The PQMB paved some streets in two neighborhoods, which participants found improved mobility and reduced falls. These PQMB improvements indirectly created an expectation of added value to local houses.

Regarding the aspects participants deemed the PQMB had overlooked, improving deteriorated sidewalks or streets emerged often in persons’ accounts, an aspect it barely included in its actions. In some neighborhoods, groups consuming alcohol or committing vandalism use such remodeled spaces (parks and multipurpose fields) constitute an outcome the program failed to foresee. On the other hand, we found few indications of coordination with other actors such as health, education,or aid agencies. Further, the construction of a park displaced the installation of a traveling vegetable market which supplied a neighborhood in Arica and generated informal sources of work. Finally, excluding houses as an object for improvement or, if impossible, directly coordinating with public entities which address this need constitutes another element straining the comprehensive nature of PMBQ interventions, ignoring that such improvements are contingent to residents’ individual resources.

### b) Psychosocial constraints (Charts 4 and 5)

**Chart 4:** Contributions of the “Quiero mi Barrio” program to individual and collective psychosocial well-being.

Perception of the neighborhood or community	Feeling of achieving community projects or solving problems diagnosed by the community	“It was a very dangerous path because it was deserted and very narrow; people drank there and threw garbage. Then, we ourselves started to want to take care of it, to fight, because it is a physical space where you can cross to the other side and so we took it as far as the footbridge” (Older lady, Barrio La Isla)
		“When we had recently arrived in the neighborhood, it was covered in stones, it was terrible (…) Now the neighborhood has changed a lot, one hundred percent, and so the children went out to play ball not like the teens could, now there’s a field, like there should be” (Older lady, Barrio Ignacio Serrano)
Participation	Temporary neighborhood participation	“Ever since we stopped doing those activities in those years, nothing was done, nothing. No one came to visit us either, nobody worried about these surroundings, nothing. Until “Quiero mi Barrio” came and things have got going again… I don’t know, neighbor meets neighbor, goes to a meeting, so things get done, I’ve… regaining the worst spaces where suddenly garbage was being tossed, that is how it was reactivated” (Leader, Barrio Lo Velásquez Norte)
		“...the only project we did is called neighborhood improvement. When we started to plant everything around, we planted a lot of little trees around the canals...it was really nice. the plants we planted, many dried up and were so beautiful, now there are one or the other... there is almost nothing, we work uselessly, and there are few of us who work on that... (Older lady, Barrio Pulmahue)
	Activation/maintenance leadership roles	“We are done with the program but we first had to pave a lane and then they started on the street in the back, another one over there, there were three lanes that were paved after us (…) neighbors came together and applied for the paving projects. (Leader, Barrio Lo Velásquez Norte).
		“We were in permanent meetings for three years, meeting after meeting. There were processes of citizen participation, citizen consultations, there was a democratic election where everyone voted knowledgeably, where the two neighborhoods voted. To elect the president and the board for the neighborhood council, and I won” (President, Neighborhood Association, Barrio La Isla)
Support networds	Creation of conditions to support families	“All kinds of people used them because there are grandparents like me who come with their grandchildren, then we go out with our grandchildren to play because their parents are busy (…) Then, young people come, teens or little ones and their grandparents (Artisans, Barrio La Isla)
		“I go to exercise, I go with my little granddaughter, she goes over to the swings. The square is pretty. (…) I had no idea and I have been going there for like three weeks” (Older lady, Barrio Ignacio Serrano)
	Spaces in which neighborhood groups and families can socialize and undertake activities	“Before… only at home. Yes, we look forward to Saturdays. We feel 10 years younger when we come here for a while (…) it is the most essential thing we’ve had. (Group of older adults, Barrio La Isla)
		“And we say that the community center is our jewel and they delivered it. We have had wakes, we celebrate Christmas, birthdays. We always ask for a contribution for its upkeep, but it is our community center with lighting and water” (President, Neighborhood Association, Barrio Bellavista)
	Use of community centers by other institutions	“Because we (the center) take care of everything, we have groups of women, we also have talks about health, (…) And we did a workshop on the subject of medicinal herbs (by CESFAM) and issues, doing courses on sewing, painting, handicrafts, so we use the center a lot” (President, Artesans, Barrio La Isla)
Security	Feeling of security	“(The lights in) the square are on, I never turn them off, we pay a lot for electricity but it doesn’t matter (…) Before, I would turn the lights on for a while and switch them off afterward but I realized that no, when they’re on, there isn’t so much mischief.” (President, Neighborhood Association, Barrio Ignacio Serrano)

**Chart 5:** Psychosocial factors negatively associated or pending with the Quiero mi Barrio program.

	Global phenomena (Cross-sectional)	
Perception of the neighborhood or community	Individual ways of life and neighborhood social life in decline	“So, we get together a bit but others aren’t interested because there are a lot of professional people or who are becoming professional here and they get home from work and shut themselves in to watch TV, the soaps, their stuff and they don’t like to go out, and they say “no, I don’t want to go out, I’m tired” (President, Neighborhood Association, Barrio Bellavista).
		“That was being lost over time for different reasons (given by the interviewees): changes on the board that prioritize other activities; low participation by locals; fewer children; many irregularities in the neighborhood association; they get together just to gossip… and as a result locals don’t participate” (Mother of a preschooler, Barrio Bellavista).
	Low participation motivated only by individual (rather than collective) interests	“When there are group activities, we invite people and they come; it’s difficult for locals to get to the neighborhood association, meetings, and events (QMB). At this point, in terms of organization, it’s difficult for them, 5, 7 but no more than that” (Leader, Neighborhood Association, Barrio Bellavista)
		“Improvements… yes they are there, people participate because they know what they’ll get, that they’re taking part because they’re going to get a benefit that’s going to be left behind for them regarding participation in “Quiero mi Barrio” (Leader NDB Barrio Lo Velásquez)
	Leadership fatigue, migration, and generational change	“We were very close, ma’am, very close… yes but not now, I see that it’s difficult (to get organized to make demands such as improving sidewalks) because older people are not here (…) Young people are already dedicated to other things, to their work, to raising their children, to fixing their houses up, each doing their own thing” (Group of Older Adults, Barrio La isla)
		“Here most older adults can’t go out because they take care of their grandchildren and I think that’s a problem” (Older lady, Barrio Lo Velásquez)
	Feeling of insecurity due to crime	“For all its lighting (the neighborhood) it’s not safe anywhere, for example, over there, behind Bulnes (…), there is a field that’s always… you can’t go there because it’s full of cars, drug dealers (…) so you can’t walk there” (President, Neighborhood Association, Barrio Ignacio Serrano)
		“The problem of drugs and its consequences (robberies, shootings, and feelings of insecurity) have caused locals to change their way of life and they are more “closed in” in their homes, without going out much and with no connections to their neighbors. The same houses have been closed in with walls and bars so people are more protected” (Youth, Barrio Lo Velásquez Norte).
	Associated with the PQMB	
	Potential reinforcement of historical unrest in neighborhoods	“They continued with ‘they are from that sector and they have this’ so there was no coming together, only between words and papers for the project. When somebody doesn’t want to work with someone else, you can’t force it. We are very close (Pob. C. Henríquez), and if nothing happens, everyone is quiet in their homes but if something happens, we are all united and there is solidarity and care for locals” (Artisans, Barrio La Isla);
		“Ok, I knew about the PQMB, but settlements have a certain dynamic of boards, meaning that there has always been conflict between boards, they are pretty strong, so I think that has made participation difficult and it will continue being difficult” (Member NDB, Barrio Bellavista)
Participation	Abrupt end to the program	“What I’m seeing is that maybe the program ends very abruptly; because if it is a program that maybe should be extended until it can be seen, I mean, a program can’t end until the community feels organized to administer that (…) Like, they create the issue and they go away. And that is probably something that should change” (Member NDB, Barrio Bellavista)
	Social groups which feel excluded or invisible	Older adult groups with medical conditions: “Yes, yes. But you don’t attend for your age, the meetings they hold, you’re on the outside of almost everything, of participating in everything. So, you stay home (…) and that is due to the climate we have too, because when winter comes (…) you don’t go out and get sick” (Group of Older Adults, Barrio Bellavista)
		Teen girls: “…Yes, because it was uncomfortable, I got there with my sister and it was like they [the boys] thought they owned the field and it was awful. After they left and it was just for the two of us, …Yes, it was awful” (Youth, 21 years, Barrio Bellavista)

Interviewees claimed feeling satisfied with the aesthetic of the neighborhood after the promoted changes and with achieving a positive outcome after the end of a process which took years of effort to improve their neighborhoods. At the time PQMB interventions begin, most neighborhoods face great housing insecurity and locals feel abandoned. Residents note that its arrival helped “to promote the self-esteem of the community” after they had experience scant support toward neighborhood improvements. Some locals value the direct PQMB actions which reinforced neighborhood memory and identity. All neighborhoods showed a drive to participate in common activities, although limited to the scope of the program. Interviewees also point to the mobilization of local leaders, who gave rise, in certain cases, to a type of organization which followed the PQMB and felt motivated to take care of the interventions and create new projects. The most common element across neighborhoods relates to the use of parks and squares — which promoted mother-children and grandmother-grandchildren coexistence in open spaces — and the benefit multipurpose fields brought to young people, men, and those who do sports. The improvement of community centers contributed to socializing, above all, older adults, housewives, and caregivers, who referred to these spaces as affecting positive their mental health. Moreover, after neighborhoods were improved and cultural centers, built, community centers began to be used for private family events (e.g., marriages, birthdays, etc.). Participants valued the aforementioned lighting of streets and public spaces as it fosters a feeling of security in neighborhoods suffering from crime, and drug trafficking.

The overlooked or limiting aspects related to program interventions include the permanent tension between the aims of the PQMB and pre-existing neighborhood dynamics. This dimension cuts across all our studied cases. We found historical conflicts between locals or leaders, socioeconomic differences, dangerous sectors within neighborhoods (e.g., drug abuse or trafficking), and a style of social leadership which sectorized actions. PQMB interventions were unable to alleviate these issues which seriously affected participation, space and constructed work use, security, and trust among locals. Neighborhoods perceived as highly vulnerable socially seem to receive no different management or treatment from those free of this connotation.

On the other hand, social life within neighborhoods (established decades earlier) is contrasted with current practices and lifestyles which exclude or reduce community life. This phenomenon is based on elements such as interests for individual benefit, more intense working lifestyles, residents’ aging, leaders’ fatigue, inhabitants’ migration, and young people’s demotivation.

In a more direct relation with PQMB management, locals from all neighborhoods recount the abrupt end of interventions, leaving a feeling of dissatisfaction among their leaders. We should point out that the program operates by creating a previously absent local organizational structure (the Neighborhood Development Board), which, although incorporating local leaders, involves coordination efforts among leaders who may be in conflict or unprepared. Some residents note that the arrival of the PQMB shifts previous dynamics of neighborhood empowerment to the improvement of spaces.

In some cases, the nature of the works has also caused a feeling of exclusion or self-exclusion in some groups (such as in older adults and young women) during the selection process for works or the type of work to be undertaken. Neighborhood insecurity (associated with crime and drug trafficking) often represents a determining element in residents’ decision to live their lives inside their homes, avoiding public spaces in the neighborhood and, therefore, their neighbors.

## DISCUSSION

PQMB is a program that focuses on improving local urban infrastructures by recovering or socially reinforcing, in a very short timeframe, the trajectory of neighborhoods and locals’ interpersonal and cultural experiences. Thus, we must interpret these experiences, adjusting them to social realities and the history of these neighborhoods, most of which emerged from past social housing programs and, in some cases, under processes of settlement and relocation.

Note that, despite differences in geography, climate, and social fabric, the works performed in the neighborhoods are similar, with only a few distinctive features due to their co-designing strategies. Nevertheless, participants generally have a positive perception of these interventions: a new or improved infrastructure represents neighborhood encounters in several places in which state aid has been absent for decades^16^. This infrastructure brings tremendous potential to reinforce and expand access to sport and recreational spaces so neighbors and relatives can coexist and socialize, infusing social organization with a certain dynamism. The aspects we mentioned function as elements which contribute to promote individual and collective health – and in some cases – restore people’s dignity.

Although no previous studies in health in Chile have addressed this frame of action, population studies^17,18^ emphasize the importance of neighborhood contexts, finding them as relevant to their inhabitants’ psychosocial experiences. International studies on processes of “urban regeneration” in Europe show beneficial results in individuals’ mental health and perceived well-being. Interventions facilitate “healthy” spaces favoring walkability, social integration, and a perception of security, promoting residents’ social and physical functionality and overall well-being, including the aesthetic function of neighborhoods^19,20^. Based on these international experiences, interventions structure leaders, promote social participation for some groups, and build larger support networks, beneficial results which enable research to understand the logic of interventions and involved social groups.

However, we find it difficult to compare experiences from approaches us used in Europe and North America to those which interest since the former usually emerge from large projects to produce public-private urban spaces. According to Paquette 2020, what defines the most typical experiences in Latin American are processes of comprehensive, multipurpose, and heavily territorialized urban improvement^21^ which are directly related to solving informal settlement problems and — in the case of Chile — to remedy the consequences of cities growing under spontaneous, informal urbanization to their outskirts and creating emergency social or subsidized housing projects which lacked a comprehensive view of individual and collective living, in which organized neighborhood communities and municipal mediation have driven a large part of the implemented improvements.

Our results show that the PMBQ has tried to strengthen neighborhood identity, driving direct actions toward it or following strategies to co-design interventions with community actors. However, it can also force actions which strengthen neighborhood individualization, fragmentation, or sectorization. This observation follows a previous evaluation of the PMBQ in twelve neighborhoods five years after its implementation^22^ which found a risk of fragmenting neighborhoods even further, as those urban projects were independently tackled and lacked public space networks (e.g., health, education, recycling, nutrition, among others).

In all studied cases, violence, crime, and vandalism emerge as latent elements causing fear, insecurity, and unrest at different levels according to each territorial context, an issue which studies in urban segregation in Chile^23^ had already underscored. It also finds itself in large-scale urban regeneration projects in Catalonia^24^ Colombia^21^, Brazil^25^, and Mexico^26^, among others. Thus, the effects of a program that works from such a noticeable focus (which in principle may be necessary) can obscure the fact that other public policies are in motion or absent, giving rise to processes of care-desattention and neglect. Thus, spaces which more proactive aims regenerated begin to reflect the uncertainties and concerns of life (e.g., homeless groups, drug trafficking, etc.). Perceived insecurity and fear configure phenomena which are closely linked to anxiety and behaviors of avoidance or confinement, negatively affecting social interaction and physical activity^27^ to the point that an urban regeneration program, even if it focuses on micro-communities, requires the action of other sectorial actors for comprehensive improvements. In health, this should foster an intersectional concept for such programs, a key issue to recognize the contributions and absences on all planes of the community life in neighborhoods^28^.

In Chile, current health promotion programs incentivize the use of community spaces in neighborhoods^29^, which is why coordination with other actors (such as those in primary care) is urgent and necessary. This study found actions promoting the spaces PQMB regenerated and the management it developed. Neighborhoods and house ownership assume a symbolic meaning of belonging (in addition to other factors conventional epidemiology has highlighted), associated with improvements on thermal comfort, noise reduction, and general satisfaction with home improvements, providing benefits for individuals’ overall, physical and mental health^30^, an aspect which can acquire important nuances depending on the ecologies of national territories.

Youth’s lack of participation in social activities and older adults feature among program actions and the attention they paid to other global phenomena. National studies in health show that more active older adults adequately channels their free time and contributes to a better quality of life, especially in contact with peer groups^31^. We must consider that care tasks hinder their participation, an increasingly frequent phenomenon derived from parental difficulties within their working-class role in society, which demands balancing family life and work^32^. This study found recurring references to older adults using spaces as they took care of their grandchildren in contrast to the lack of spaces for themselves, raising the need for working perspectives which include the individual and collective interests and developments in this predominant group within the projects regeneration of neighborhoods.

We found that the creation of sport spaces promotes younger groups’ practice and relaxation, positively affecting their physical and mental health^33^. This study evinces that these interventions influence the emergence of these conditions. However, the studied neighborhoods show no new sport groups, rather, recurrent user groups deemed that the conditions for their activities had improved. On the other hand, the strong PQMB direction to enable spaces for children’s sports and recreation ignores other, woman youth or teen mothers’ interests for whom these spaces, although valued, are yet to be deemed as spaces which might attract users. This represents an opportunity for the program to consider different needs and experiences within neighborhoods.

Among our limitations, we failed to find groups who had organized themselves around an experience continuum, only attesting to actors who already were participants and familiar with certain neighborhood changes. Thus, key informants predominate in this study. We were also unable to further develop research with more focus groups in some neighborhoods since researchers felt endangered by doing so, requiring a greater number of individual interviews.

The strengths of this study include assessing collective health and well-being to address urban regeneration as a local scenario requiring public health perspectives. Our sample also highlights the participation of various geographic areas and relatively recent experiences. Given the evidence this study has provided and considering the guidelines of the SDH approach, primary care and local community team actions represent an opportunity to both design and implement the PQMB.

## CONCLUSIONS

Locals perceived that the urban changes arising from the PQMB improved the infrastructure and psychosocial surroundings of several neighborhoods. Participants deemed these beneficial changes as promoting collective well-being, listing its socialization and walkability spaces, support networks, lighting, cleanliness, and public security the main contributions of the program. Nevertheless, they perceive global phenomena as associated with insecurity, inequity, aging, and new lifestyles, in addition to the aspects the program overlooked, which limit its reach and affect locals’ perception of well-being. Within the SDH approach we adopted, i.e., spheres of direct effect, the structural aspect represents a public policy, operationalized via the PQMB, which has the possibilities and opportunities of receiving praise for its benefits, coverage, and methods of implementation. These mechanisms require further development to analyze the temporality of the interventions and, above all, highlight the importance of planning and intersectoral work to pursue comprehensive actions in urban spaces.
